# Cohesion-driven mixing and segregation of dry granular media

**DOI:** 10.1038/s41598-019-49451-z

**Published:** 2019-09-17

**Authors:** Ahmed Jarray, Hao Shi, Bert J. Scheper, Mehdi Habibi, Stefan Luding

**Affiliations:** 1 Multi-Scale Mechanics (MSM), University of Twente, NL-7500 AE Enschede, The Netherlands; 2 Laboratory of Physics and Physical Chemistry of Foods, Wageningen University, Wageningen, The Netherlands

**Keywords:** Soft materials, Mechanical engineering, Chemical engineering

## Abstract

Granular segregation is a common, yet still puzzling, phenomenon encountered in many natural and engineering processes. Here, we experimentally investigate the effect of particles cohesion on segregation in dry monodisperse and bidisperse systems using a rotating drum mixer. Chemical silanization, glass surface functionalization via a Silane coupling agent, is used to produce cohesive dry glass particles. The cohesive force between the particles is controlled by varying the reaction duration of the silanization process, and is measured using an in-house device specifically designed for this study. The effects of the cohesive force on flow and segregation are then explored and discussed. For monosized particulate systems, while cohesionless particles perfectly mix when tumbled, highly cohesive particles segregate. For bidisperse mixtures of particles, an adequate cohesion-tuning reduces segregation and enhances mixing. Based on these results, a simple scheme is proposed to describe the system’s mixing behaviour with important implications for the control of segregation or mixing in particulate industrial processes.

## Introduction

Mixing of granular materials is important in several particulate processes such as concrete preparation, chemical formulation and pharmaceutical engineering^1,2^. Under the presence of shear, granular materials in these processes often segregate owing to differences in particle properties such as the size and density, causing them to sink or rise within the bed and to eventually accumulate, leading to a variation of the bulk composition and density^3,4^. This segregation behavior can be a difficult problem in some processes that may critically degrade the quality of the final product (e.g., solid dosage forms preparation and food processes), and, in some other cases, can be used for benefit (e.g., recycling and separation processes). Thus, it is important to understand particulate segregation mechanism to efficiently prevent, trigger, enhance or reduce it.

Among the several size segregation mechanisms^5^, the kinetic sieving is the dominant mechanism causing segregation in gravity-driven free-surface flows^6,7^, where the small particles move effectively, shifting downwards, as they are more likely to move into the voids between larger particles. This mechanism performs well when the volume fractions of large and small particles are equal^8^. Using numerical simulations, Hong *et al*.^9^ considered the interplay between size and mass in a binary mixture of spherical particles, and predicted the existence of the reversed Brazil Nut effect (RBN). Khakhar and co-workers^10^ showed that the segregation rate of dry particles is independent of the filling level of the rotating drum. Alonso *et al*.^11^ investigated the segregation of particles due to differences in size and density in a two-dimensional horizontal rotating cylinder, and proposed an expression of segregation index to predict if a large particle tends to float or sink in a bed of fine particles. More recently, Hongyi *et al*.^12^ applied unsteady flows to strongly segregating granular materials in an attempt to control the segregation pattern and enhance mixing.

A typical approach to prevent size segregation is by making particulate components sizes as close as possible^13^, or by using cohesive particles^14^. However, cohesion between particles is difficult to introduce and control. As a consequence, several studies regard particles as non cohesive^15–17^, but cohesion is always present in most industrial particulate processes, and thus has to be taken into account. In some other studies, cohesion was introduced using various viscous liquids with different capillary forces^14,18–21^. Jarray *et al*.^19^ investigated the effect of liquid induced cohesion on granular flow in a rotating drum and showed that capillary cohesion increases the angle of the slope and decreases the granular temperature at the free flowing surface. Shi *et al*.^22^ performed experiments on various size fractions of cohesive limestone powders and showed that cohesion dominates the flow behaviour for fine particles. Li and McCarthy^14^ showed that segregation in granular systems can be controlled by adding moisture. Roy *et al*.^23^ studied the effect of wet cohesion on the granular flow and proposed a rheological model that predicts various features such as the shear thinning behavior. Chou *et al*.^24^ demonstrated that the segregation index in wet granular materials decreases with an increase of the angle of the slope in a rotating drum, regardless of the volume or viscosity of the added liquid. Apart from studies on segregation in wet systems with cohesion due to capillary forces, there are less experimental investigations focusing on dry cohesive systems, especially on studies where the cohesive force can be adjusted.

One way to modify the glass surface and obtain dry cohesive particles is the so-called silanization. The original purpose of chemical silanization is to change the hydrophobicity of glass surface^25^. Here, we use extensive silanization to modify the contact cohesion of millimetric size glass particles in a controlled way, with the goal to link this micro-cohesion change to the macroscopic flow and mixing behavior. In industry and within the scientific community, several geometries are used for particulates mixing^1,4,26,27^. In this study, we employ a rotating drum to study the flow and segregation of cohesive and non-cohesive granular materials. This apparatus is extensively used in experiments and as a model system due to its simple geometry comparing to other mixing devices.

To our knowledge, this is the first paper that experimentally investigates the effect of adjustable cohesive forces on the segregation of dry granular systems in a rotary shearing device. We characterize the cohesive particles properties using different experimental approaches, which include Atomic Force Microscopy (AFM) for surface scanning of the particles, bulk flowability in the drum, and cohesion force measurement using an in-house setup. The effects of silanization reaction duration on the surface properties of the particles are presented, and the cohesive and adhesive forces between particles are measured and discussed. Then, cohesive particles are mixed with non-cohesive particles in a rotating drum mixer, and the segregation is investigated under different combinations of cohesive forces and particle sizes, concluding that the cohesion-dependency of segregation may be manipulated to mitigate or enhance particulate mixing.

## Results and Discussions

### Characterization of the surface and cohesive properties of the particles

**Figure 1:**
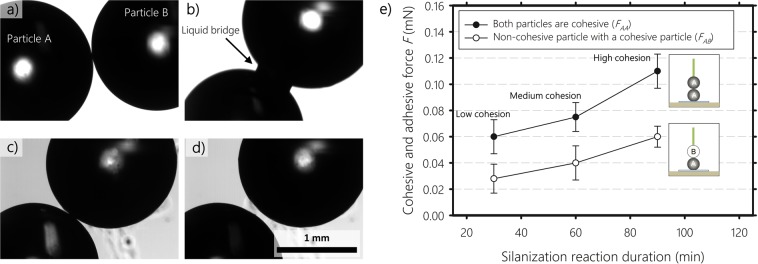
Microscopic observation of glass particles of radius 0.85 mm, (**a**) Dry untreated particles, (**b**) Wet untreated particles, formation of a liquid bridge, (**c**) Dry silanized particles in contact, (**d**) Dry silanized particles separated by a small distance to show the absence of a capillary bridge, and (**e**) Cohesive and adhesive forces between two particles of radius 0.85 mm as a function of the silanization reaction duration.

Typically, to investigate the effect of cohesion on particulate segregation and flow, researchers use capillary forces^28–30^. When particles are in contact with each other in the presence of a small amount of liquid, the interstitial liquid forms a liquid bridge between the particle and the cohesion is dominated by capillary forces (see Fig. 1(b)). This approach has several limitations. For instance, the liquid distribution, and hence capillary force distribution, within the particulate system is not uniform especially under dynamic conditions^31,32^. Additionally, tuning the capillary force is not straightforward as it depends on both liquid and particulate properties. To avoid these complications, we use silanization where cohesiveness is only due to immediate chemical bonding with no formation of capillary bridges. In this case, cohesion between particles is only upon contact. If the particles do not touch, the cohesion force is equal to zero due to the very short range of the attractive force. This can be seen in Fig. 1(c,d), where silanized particles are perfectly dry and capillary bridges are absent, making their cohesive behaviour as close as possible to that of real dry cohesive powders.

Figure 1(e) shows the cohesive (*F*_*AA*_) and adhesive forces (*F*_*AB*_) between two particles of radius 0.85 mm as a function of silanization reaction time. Here, “A” particles are cohesive and “B” particles are non-cohesive. The cohesion and adhesive forces increase with silanization reaction duration. The adhesive forces between non-cohesive and cohesive particles are order of half of the cohesive force between two cohesive particles. Highly cohesive particles of about 0.11 mN are obtained when the silanization reaction duration is longest (i.e., all the Heptane in the Silane solution evaporates during the silanization process). This increased cohesive force can be attributed to the formation of more covalent bonds (-Si-O-Si-) as the reaction progresses.

**Figure 2:**
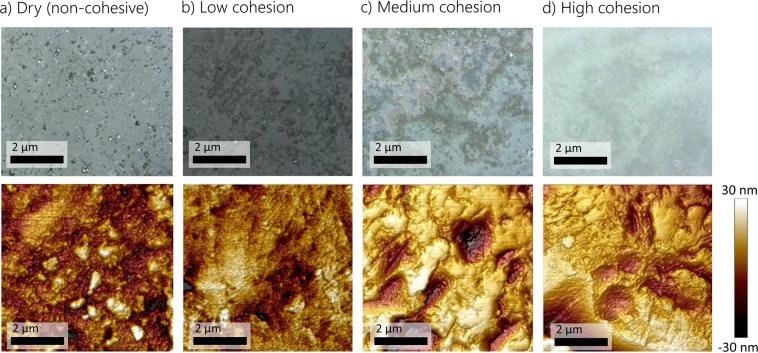
Microscopic scanning (top) and AFM measurement (bottom) of (**a**) dry non-silanized, (**b**–**d**) silanized particles.

Figure 2 shows microscopic and AFM measurements of dry and silane treated glass particles. Cohesive silanized particles seem to have smoother surfaces with less irregularities than non-silanized particles. Less irregularities infers a more uniform coating, and hence is an important factor in determining the cohesive force between particles. Since the thickness of the Silane coating is in the range of 30 nm, it is assumed that the main elastic properties of the individual particles are still the same after silanization.

**Figure 3:**
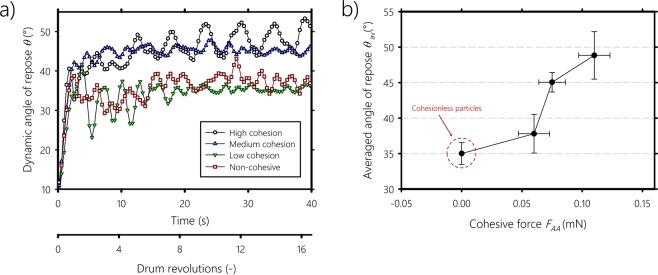
(**a**) Smoothed dynamic angle of repose as a function of time for dry and cohesive particles of radius 0.85 mm, (**b**) Averaged dynamic angle of repose as a function of the cohesive force after 20 seconds of drum rotation.

In addition to direct cohesive measurements, the cohesive force can be correlated with the angle of the flow (i.e., dynamic angle of repose) at the bulk level in a rotating drum. We show in Fig. 3(a) the smoothed angle of the flow *θ* in a rotating drum as a function of time for non-cohesive and cohesive particles. The particles are of radius 0.85 mm and the rotation speed of the drum is 25 rpm (i.e., Froude number *Fr* = 0.21). As the drum rotates, particles are lifted to the upper part of the bed and the angle of the slope increases until it reaches a maximum, then avalanches start to occur. For dry particles, successive avalanches happen with large amplitude followed by a relatively steady flow with small avalanche amplitude variations. For highly cohesive particles, successive avalanches remain as the drum rotates. This is because cohesive forces dominate the flow and particles become closely packed and start to flow as a bulk. For medium cohesive particles, the amplitude of the avalanches is the lowest indicating a liquid-like flow behavior. When the cohesion is low, the dynamic angle of repose is close to that of the non-cohesive case. Figure 3(b) shows the dynamic angle of repose averaged over the last 20 seconds versus the cohesion force. The dynamic angle of repose increases with the cohesive force, qualitatively confirming the results obtained by the microbalance setup in Fig. 2. By analogy to the flow of wet particles in the presence of capillary forces^18,19^, we can infer that the increase of the dynamic angle of repose is because the cohesive forces prevent the rolling and cascading of individual particles in favor of bulk sliding. These experiments show that the cohesive force between particles can be adjusted using the silanization reaction duration, and can also be characterized at the bulk level using the angle of the flow in a rotating drum.

### Particles segregation

Here, we investigate the effect of the cohesive force on particles segregation. We conduct a set of experiments with different particle sizes and different cohesive forces as shown in Table 1. In all cases, the drum contains a mixture of 50-50% by weight (w/w) (i.e., 50% of particles “A” and 50% of particles “B”) glass particles with the same density of 2500 kg/m^3^.

**Table 1:** Cases used for the segregation experiments.

Cases	1	2	3	4	5	6	7	8	9	10	11	12
Radius *r*_*A*_	0.85	0.85	0.85	0.85	0.85	0.85	0.85	0.85	0.85	0.85	0.85	0.85
Cohesion *F*_*AA*_ (mN)	0	0.06	0.075	0.11	0	0.06	0.075	0.11	0	0.06	0.075	0.11
Radius *r*_*B*_ (mm)	0.85	0.85	0.85	0.85	1.25	1.25	1.25	1.25	2	2	2	2
Cohesion *F*_*BB*_ (mN)	0	0	0	0	0	0	0	0	0	0	0	0
Adhesion *F*_*AB*_ (mN)	0	0.028	0.04	0.06	0	0.04	0.08	0.12	0	0.067	0.11	0.17

**Figure 4:**
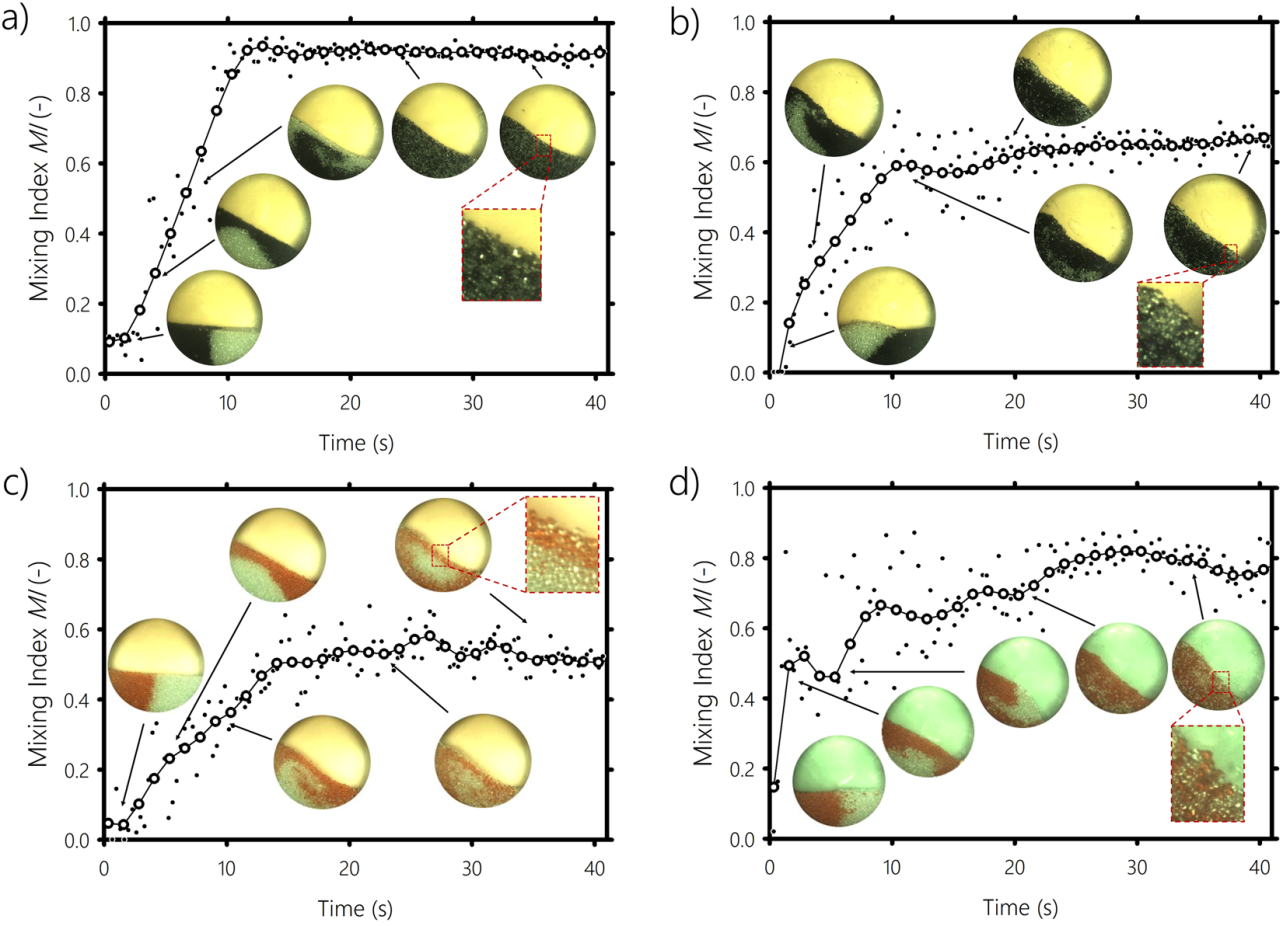
Mixing index as a function of time for: (**a**) Non-cohesive system, case 1, (**b**) Cohesive system, case 4, where both black and transparent particles have the same size of 0.85 mm, (**c**) Non-cohesive system, case 5, (**d**) Cohesive system, case 8, where the transparent particles radius is *r*_*A*_ = 0.85 mm and the red particles radius is *r*_*B*_ = 1.25 mm.

We plot in Fig. 4(a,b) the mixing index for monosized particles, of radius *r*_*A*_ = 0.85 mm, as a function of time for cohesionless (case 1) and cohesive particles (case 4), respectively. The mixing index curves are smoothed using bisquare weighting smoothing to reduce the fluctuation of the mixing index. The raw values obtained directly from image post-processing, before smoothing, are shown in dots in Fig. 4 and in the Supplementary Information file, Appendix A. A Mixing Index (*MI*) close to 1 means the system is mixed, while an *MI* value close to 0 indicates a segregated system. The mixing index in Fig. 4(a) increases at first and then, after few rotation of the drum, remains relatively constant at around *MI* = 0.9, indicating good mixing of the system. However, as shown in Fig. 4(b), when the transparent particles are cohesive, a lower *MI* value around 0.6 is obtained and segregation occurs between monosized black and transparent particles. This can be explained by the clustering of the transparent particles by cohesion, which pushes them outside and keeps the black particles within the core region of the bed, where the flow is quasistatic. By performing Discrete Element Method (DEM) simulations of a mixture of cohesive and non-cohesive monosized particles in a rotating drum, Yazdani and Hashemabadi^33^ observed similar behaviour, where the granular system tended to segregate and cohesive particles moved towards the outer layer of the rotating granular media.

We show in Fig. 4(c) the mixing index (*MI*) versus time for a bidesperse system composed of non-cohesive transparent particles of radius *r*_*A*_ = 0.85 mm mixed with non-cohesive red particles of radius *r*_*B*_ = 1.25 mm (case 5). In contrast with the previous case, when the system is composed of bidisperse particles, after few rotations of the drum, the mixing index reaches a steady state at about 0.5, indicating segregation, where larger particles are found in the upper region of the bed and near the wall of the drum, and smaller particles are concentrated in the core of the bed surrounded by the large particles. When the same experiment is performed with cohesive particles, the results are different. Figure 4(d) shows the mixing index (*MI*) of case 8, where particles of radius *r*_*A*_ = 0.85 mm are cohesive and mixed with non-cohesive larger particles of radius *r*_*B*_ = 1.25 mm. The value of the mixing index in this case is higher than for case 5, indicating better mixing with fewer large particles found in the outer region and more of them in the core of the bed. This demonstrates the ability of cohesion to improve dry particulate mixing. The cohesive force clumps the small particles together, decreasing their probability of moving into gaps between larger particles, which is similar to the explanation given by Li and McCarthy^14^ for the case of cohesion due to moisture between the particles.

**Figure 5:**
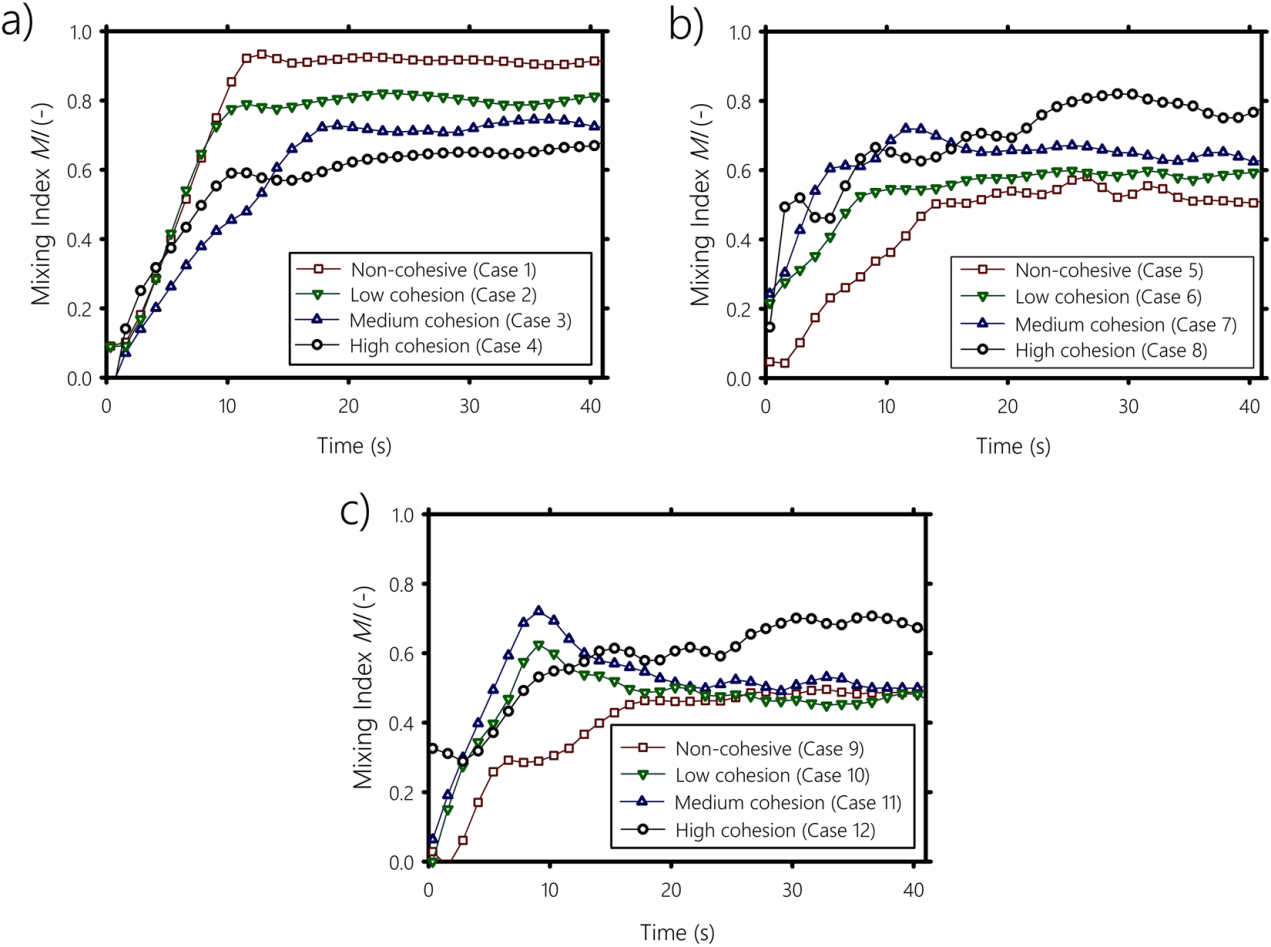
Mixing index against time for different cohesive forces and various combinations of particle sizes. (**a**) Monosized particles of 0.85 mm, (**b**) Combination of 0.85 and 1.25 mm particles radii, and (**c**) Combination of 0.85 and 2 mm particles radii.

We report in Fig. 5 three plots of the mixing index measurements for all the cases described in Table 1 as a function of time. In Fig. 5(a), we show cases 1 to 4, which correspond to systems where the particles have the same size. In all four cases, the mixing index reaches a steady state after approximately 7 revolutions of the drum (i.e., 20 seconds). The final mixing index value decreases as the cohesive force increases, confirming that cohesion reduces mixing. We also notice that the higher the cohesiveness, the more time is needed by the system to reach the steady state. Figure 5(b) shows the mixing index for a system composed of particles of radii *r*_*A*_ = 0.85 mm and *r*_*B*_ = 1.25 mm for different cohesive forces (cases 5 to 8). Again, after few rotations of the drum, the mixing index curves reach a plateau. When the cohesion is low (case 6), *MI* reaches slightly higher values than in case 5, where particles are not cohesive. As the cohesive force increases, segregation reduces, indicating that cohesion plays a key role in improving the mixing of particulate systems through the formation of clusters. We infer that cohesion makes small cohesive particles to clump together impeding them to pass through the gaps between the large ones. By performing DEM simulations, Aarons *et al*.^34^ arrived to the same statement. They found that the tendency for the small particles to form agglomerates and their ability to move between the big particles are the main mechanisms that control cohesive particulate mixing. Since the cohesive small particles ability to mitigate segregation is dependant on the size of the large particles, we carried out the same experiments but with larger non-cohesive particles of radius *r* = 2 mm for different cohesive forces (cases 9 to 12). The mixing index for these cases is shown in Fig. 5(c). Similarly to Fig. 5(a,b), a plateau is observed after 20 seconds of drum rotation. For the non-cohesive system (case 9), the value of the mixing index at the plateau is about 0.48, slightly lower than the value obtained in case 5 (i.e., non-cohesive combination of 1.25 mm and 0.85 mm), which is in accordance with the findings of previous work^24,35–37^, stating that a greater difference in size of cohesionless particles increases the degree of segregation. After 7 rotations of the drum, low and medium cohesive particles (cases 10 and 11, respectively) reach almost the same level of mixing as the cohesionless case (i.e., case 9), indicating that the cohesion is not strong enough to prevent 2 mm particles segregation. Only highly cohesive particles (case 12) are able to reduce 2 mm particles segregation. All these results show that the mobility of the non-cohesive particles depends strongly on the cohesive force between the particles, which, more importantly, can be tuned to control segregation.

**Figure 6:**
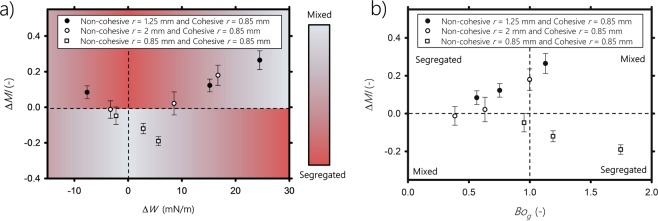
Mixing index resulting from cohesive forces $\Delta MI=M{I}_{coh}-M{I}_{non-coh}$, versus (**a**) cohesion work change Δ*W*, (**b**) the granular Bond number *Bo*_*g*_.

Next, we present diagrams to visually identify zones where systems tend to mix or segregate. We plot in Fig. 6(a) the mixing index resulting from cohesion, $\Delta MI=M{I}_{coh}-M{I}_{non-coh}$, averaged over the last 10 seconds of the duration of the experiment, as a function of the cohesive work change (i.e., surface energy change per particle) when the system goes from a segregated to a mixed state $\Delta W={W}_{m}-{W}_{s}$, with *MI*_*coh*_ and *MI*_*non*–*coh*_ are the averaged mixing index for the cohesive case and the non-cohesive case, respectively, and *W*_*m*_ and *W*_*s*_ are the cohesive work per particle of the perfectly mixed system and the segregated system, respectively. When the mixture is bidisperse (circle symbols in Fig. 6(a)), the mixing degree increases with Δ*W*, and the cohesion of the particles provides work (i.e., surface energy) that reduces the segregation of the system. Δ*W* = 0 mN/m means either *W*_*s*_ = *W*_*m*_, or all particles are non-cohesive. In case *W*_*s*_ = *W*_*m*_, the adhesive work exerted by the cohesive small particles on the large particles is counterbalanced by the cohesive work between the small particles. Either way, the system will segregate by size. This suggests that, as long as Δ*W* is sufficiently large, the mixing of the bidisperse system is enhanced. This is inline with the work of Chaudhuri *et al*.^38^, who examined computationally the effect of adhesive forces in a binary system in the absence of cohesive forces. They found that adhesion favors the mixing process. However, we expect that further increasing Δ*W* will reach a point where the system becomes perfectly mixed. Beyond this point, cohesion will start reducing the mixing of the system, because relatively large clumps of small particles, larger than the large individual particles, will form and migrate to the outer region of the bed by size segregation. This was emphasized by Sarkar and Wassgren^39^, who stated that further increasing the cohesion forces may start hindering the mixing. On the contrary, for monodisperse particles (square symbols in Fig. 6(a)), the mixing decreases with Δ*W* and cohesion induces segregation. For Δ*W* = 0 mN/m —i.e., the segregation and mixing work are balanced, the monodisperse system is well mixed.

To quantify the importance of cohesion in comparison to the weight of the particles, we plot in Fig. 6(b) the mixing index due to cohesion, $\Delta MI=M{I}_{coh}-M{I}_{non-coh}$, as a function of the granular Bond number *Bo*_*g*_. The granular Bond number refers to the ratio of the maximum cohesive force, *max*$({F}_{AA},{F}_{AB})$, to the effective weight of the particles:

1 $$B{o}_{g}=\frac{{\max }({F}_{AA},{F}_{AB})}{\frac{4}{3}\pi g{(\frac{2{r}_{A}{r}_{B}}{{r}_{A}+{r}_{B}})}^{3}\rho }.$$

Figure 6(b) shows that for bidisperse systems (circle symbols), the mixing increases with *Bo*_*g*_. When *Bo*_*g*_ > 1, the maximum cohesive force is greater than the effective weight of the particles, and mixing occurs. When *Bo*_*g*_ < 1, the interparticle cohesive forces are weak comparing to the effective weight, and thus cannot prevent segregation. We expect that when the bond number reaches a value higher than a critical Bond number $B{o}_{g}^{c}$, the bidisperse system will start segregating because the size of the clustered particles will exceed that of the large particles. We define $B{o}_{g}^{c}$ as the bond number where the maximum cohesive force is equal to the weight of the large particles, which gives $B{o}_{g}^{c}={(({r}_{A}+{r}_{B})/2{r}_{A})}^{3}$ (see Supplementary file for more details, Appendix B). Unlike the bidisperse case, for monosized particles (square symbols in Fig. 6), segregation is enhanced when *Bo*_*g*_ increases due to cohesion. Here, *Bo*_*g*_ < 1 indicates mixing. This is because cohesive forces are not strong enough to counterbalance the equally weighted particles of the perfectly mixed system. This counterbalancing occurs when *Bo*_*g*_ > 1, causing the system to start segregating due to small glass particles agglomeration. Notice that in this case, $B{o}_{g}^{c}=1$, which also indicates that cohesion will reduce mixing. This is supported by the numerical simulations of Sarkar and Wassgren^39^, who showed that cohesive interactions between monosized particles promote agglomerate formation, which decreases mixing. Similarly, Halidan *et al*.^40^ showed numerically that monosized particles mixing is better at moderate and low interparticle cohesion because the cohesive bonds between particles are easy to break. Comparing the results given by the cohesive work change approach (Fig. 6(a)) and those given by the Bond number approach (Fig. 6(b)), both give similar results, but the former seems to be more accurate in terms of predicting the mixing degree of the system.

## Summary

Using controlled chemical silanization, it was possible to generate glass particles surfaces with adjustable cohesion. The cohesive force between particles was measured using an in-house experimental device and also characterized on the bulk level using the dynamic angle of repose in a rotating drum. We determined experimentally that an increase in cohesive forces increases the dynamic angle of repose, and the smooth flow of cohesionless particles transforms into an irregular flow of a bulk of clumped particles. Then, we investigated the effect of cohesive forces on the degree of segregation in the rotary drum. While strong enough cohesion of small particles improves mixing for bidisperse systems, it causes segregation for monosized particles. Therefore, one should be able to switch between size segregation or mixing of monosized particles simply by tuning on or off the cohesive forces between the particles. For bidisperse systems, avoiding segregation requires a balance between size segregation and cohesive clustering of the small particles. Our experiments illustrate the importance of cohesion for the controlled minimization of particle segregation during large-scale processing of powdery materials, capturing the mixing dependence on particle size and adhesive/cohesive forces between particles. The developed state diagrams can be extended to other size ratios, and may serve as a useful tool for controlling particle mixing in other devices or processes (e.g., slow chute flow, slowly vibrating bed) where shearing is not so strongly localized. Additionally, it would be of great interest to investigate whether similar mixing behaviour is obtained when the large particles are cohesive. Also, it would be important to extend our analysis to other weight fractions of large and fine particles.

## Methods

### Glass surface pretreatment: Silanization process

Silanization is based on the adsorption, self-assembly and covalent binding of Silane molecules onto the surface of glass particles. Silanes are coupling agents that can interact with both organic and inorganic materials. The silanization process has been used by many scientists for the surface hydrophobization of glass and other materials^19^. It was also applied to fine silica particles as rheological additives for adhesives, resins and paints^41–43^.

Chemical compounds used for silanization are: silanization solution 5% (V/V, 5% in volume of Dimethyldichlorosilane in Heptane, Selectophore), Hydrochloric acid (HCl, 0.1 mol), Acetone and Ethanol. The procedure for making the glass particles cohesive is as follows: First, to ensure that the surface of Silica glass particles is free of contamination, they were cleaned for at least one hour by immersion into freshly prepared HCl solution under agitation using a rotor-stator homogenizer. Then, they were rinsed thoroughly with deionized water and oven dried for 3 hours at 60 °C. Afterwards, the freshly cleaned samples of glass particles were immersed in the silanization solution under low agitation speed at room temperature for various durations of time (30, 60 and 90 min) to obtain samples of particles with different cohesive forces. During this process, the inorganic functional groups of the Silane reacts with the different OH groups formed after cleaning with HCl and forms Si-OH groups. Finally, the treated glass particles were allowed to air-dry under a fume hood for 24 hours, forming a nanometric thin coating around the surface of the particle. The cohesive force of the formed coating mainly depends on the chemical silanization reaction duration and the Silane concentration.

### Cohesion force measurement

**Figure 7:**
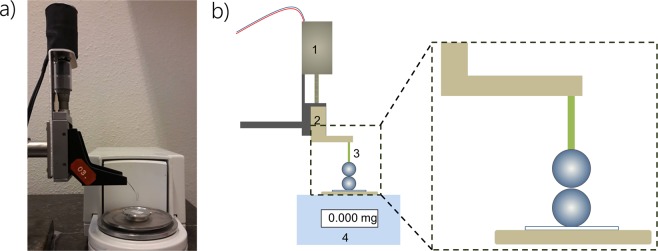
(**a**) Microbalance setup, (**b**) Schematic representation of the microbalance setup, (1) electric motor, (2) micro-positioner, (3) two particles, one on a flexible thin rod and the other one on a double sided tape, and (4) micro-balance.

The cohesive force between the particles was measured using an in-house setup that we specifically designed for this study at the Laboratory of Physics and Physical Chemistry of Foods (Wageningen University, NL). The setup consists of a micro-balance (Sartorius) and a micro-positioner as shown in Fig. 7. The micro-positioner was driven by a DC electric motor with an adjustable speed varying from 0.05 to 1 mm/min. The micro-positioner was mounted above the micro-balance. The whole setup was installed on an optical table to reduce mechanical vibrations. One particle was fixed on the balance by a double-sided tape while the other particle was glued to a flexible thin rod connected to the micropositioner.

In order to stick the upper particle to the flexible rod without contaminating the whole particle surface with the glue, the tip of the rod was first wetted with about 0.05 micro-liters of instant liquid resin. After about 20 seconds, the liquid resin became more viscous, and then the upper side of the particle was precisely put in contact with the glue. After another 30 seconds, the particle was firmly connected to the rod. In order to measure the adhesive force, first the upper and lower particles were aligned as shown in Fig. 7. The weight in the balance is set to zero, subtracting the weight of one glass particle. Then, the top particle was moved downward with a speed of 1 mm/min. Upon the first mechanical contact between the particles, the balance shows a positive weight. Then, the stage is moved upward slowly (0.05 mm/min). When the two particles separate, the maximum absolute value obtained is recorded as the cohesive pull-off force between the particles. The stronger the particle-particle cohesive force, the stronger the snap-in force. This measurement has been repeated (more than 3 times) for several particles of different sizes and cohesive forces. The ideal accuracy of the micro-balance would be about 10^−8^ mN. However, airflow around the setup and mechanical vibrations reduce the accuracy of our measurements to about 10^−5^ mN.

### Surface characterization: AFM measurement

The AFM observations were performed with a Dimension Fast Scan atomic force microscope (Bruker’s ScanAsyst and PeakForce Tapping AFM, Bruker Corporation, UK), located in the NanoLab NL, University of Twente. All AFM images were collected using the tapping mode with a silicon nitride probe and a scanning area of 2 × 2 *μ*m. The glass particle samples were glued onto a double side silica tape on top of a glass substrate before the tapping measurements.

### The drum apparatus

The drum is made by a cylinder of *R* = 60.5 mm inner radius, and *L* = 22 mm length, held between two circular Plexiglass (PMMA) plates of 5 mm thickness to allow optical access. For a quasi-two-dimensional rotating drum, Jain *et al*.^3^ argued that the length of the drum in the axial direction should be larger than 6.4 × *r*, with *r* the average radius of the particles, to neglect the front and back wall friction on the flow characteristics. The drum length used in this study is indeed larger than 6.4 × *r* of all the particles used. The drum was placed on a horizontal rotating axis driven by a variable-speed motor. Images of the rotating drum were recorded using a MotionBLITZ EoSens high speed camera working at 120 fps.

Experiments were performed in the cascading regime, at a rotation speed of the drum *ω* = 25 rpm, corresponding to a Froude number *Fr* = 0.21. This number represents the ratio of centrifugal to gravitational acceleration:

2 $$Fr=\sqrt{\frac{{\omega }^{2}R}{g}},$$

where *ω* is the rotation speed of the drum, *R* its inner radius and *g* the acceleration due to gravity. Experiments are conducted using a selected set of Borosilicate glass particles of density 2500 kg/m^3^. The drum is less than half filled with the same mass of particles (i.e., 125 g of particles). Characteristics of the drum and the glass particles are summarized in Table 2.

**Table 2:** Properties of the drum and the glass particles.

Properties	Value
Drum, *R* × *L* (mm)	60.5 × 22
Drum rotation speed *ω* (rpm)	25
Glass particles radii *r* (mm)	0.85, 1.25 and 2
particle density *ρ* (kg/m^3^)	2500
Filling level of the drum *β* (%)	35

### Image post-processing and mixing index computation

The images, acquired using the high-speed camera, were post-processed to obtain the dynamic angle of repose and the mixing index in the rotating drum. The dynamic angle of repose was obtained using the particle tracking package Trackmate within the FIJI ImageJ distribution^44^. First, we removed the background in the image sequence and adjusted the light to enhance particle detection. Particles outlines (i.e., spots) that stand out from the background were segmented and identified based on the difference of Gaussians distributions^45^ with an estimated particle diameter of 8 pixels for a particle radius of *r* = 0.85 mm. The detected spots were converted into a table of positions and visualized using ParaView^46^. The dynamic angle of repose was computed by linear regression of the positions of the particles in the free surface of the bed using an in-house python code.

For the computation of the mixing index, particles with different colors were used in the experiments to identify cohesive, non-cohesive, big and small particles during image post-processing using variable tolerances in FIJI ImageJ software. Then, binary images with different particle species were obtained and converted into continuum density fields using a method similar to Coarse Graining method (CG)^47^. The main difference between the two methods is the application of the CG to the pixel data rather than the particle positions. This fully removes the need for a particle size and position detection algorithm, leading to a significant reduction in analysis time. To obtain the density field, we assumed the mass to be equal to 1, which gives a CG density function of:

3 $${\varphi }_{S}^{i,j}=\mathop{\sum }\limits_{p=1}^{P\in S}\,\Omega ({x}_{p}),$$

with ${\varphi }_{S}^{n}$ the local density at position (*i*, *j*), summed over all particles $P\in S$, $\Omega (p,k)$ a Gaussian distribution with the mean placed on particle positions (*p*, *k*), and the standard deviation is the radius of the particles. The obtained particle density fields are then used to compute the Mixing Index *MI*. We use the mixing index based on information entropy according to Schutyser *et al*.^48^, since it was successfully used before in molecular dynamics and granular systems^48,49^. To define *MI* for two species *a* and *b*, we use the Boltzmann expression for entropy:

4 $${s}^{i,j}={\varphi }_{a}^{i,j}ln({\varphi }_{a}^{i,j})+{\varphi }_{b}^{i,j}ln({\varphi }_{b}^{i,j}),$$

Then, we calculate the system entropy by averaging the local sampled entropies as:

5 $$S=\frac{1}{N}\,\sum _{i,j}\,{s}^{i,j}{\Phi }^{i,j}.$$

Here, ${\Phi }^{i,j}$ is the local density of all particle species, and *N* is the total number of positions. The mixing index is then defined as:

6 $$MI=\frac{S-{S}_{s}}{{S}_{m}-{S}_{s}},$$

where *S*_*s*_ and *S*_*m*_ are known reference values of a perfectly segregated and mixed images of our systems, respectively. This leads to *MI* = 1 for a fully mixed system, and to *MI* = 0 for a fully segregated system.

The mixing index is then computed for all image frames of the experiment to get the evolution of the mixing state of the system. For both CG and mixing index computation, an in-house Matlab script was used. The difference between the mixing index in the presence of cohesive particles (*MI*_*coh*_) and the mixing index in the absence of cohesion (*MI*_*non*–*coh*_), $\Delta MI=M{I}_{coh}-M{I}_{non-coh}$, gives the cohesive contribution to the mixing of the system. Alternatively, if one needs to compute the corresponding entropy-based Segregation Index (*SI*), the following equation can be used:

7 $$SI=1-MI=\frac{S-{S}_{m}}{{S}_{s}-{S}_{m}}.$$

### Theoretical model of binary cohesive and adhesive systems

**Figure 8:**
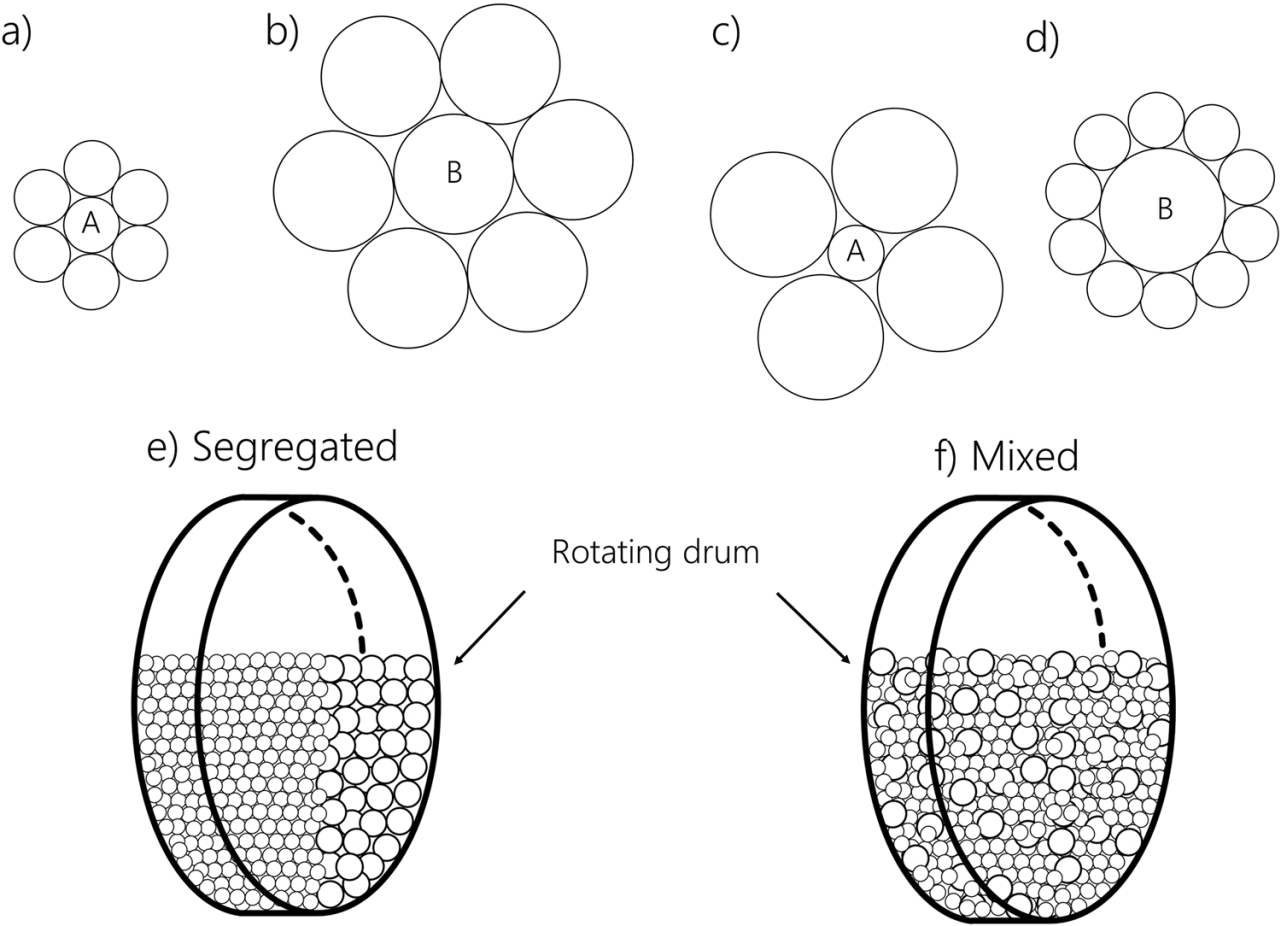
(**a**–**d**) Schematic 2D view of four special cases of contacts between particles, (**e**) perfectly segregated system of particles, and (**f**) well mixed system, both in a rotating drum.

When a cohesive particle goes from a segregated to a perfectly mixed state, it must spend a certain amount of energy in order to break the cohesive/adhesive bonds of the segregated configuration, and will acquire another amount of energy when forming new bonds in the mixed configuration. This adhesion/cohesion energy change is key for determining whether the cohesive particles will reduce or enhance segregation. The higher this energy change, the stronger the effect of the cohesive/adhesive forces on the behaviour of the system. We will derive this energy change by determining the number of contacts per particles in a bimodal system, and by calculating the work of adhesion/cohesion (i.e., surface energy) present at each contact between the cohesive particles. Let us consider a bimodal mixture of spherical particles species “A” and “B” of different radii. Four extreme cases of contact configuration are shown in Fig. 8: (a) particles “A” in contact with a reference particle “A”, (b) particles “B” in contact with a reference particle “B”, (c) particles “B” in contact with a reference particle “A”, and (d) particles “A” in contact with a reference particle “B”.

Bouvard and Lange^50^ proposed a micromodel for the prediction of the number of contacts between particles for the four cases, described in Fig. 8 in a bimodal mixture that consists of the following equations:

8 $${Z}_{AA}=3+\eta -{Z}_{AB},$$

9 $${Z}_{BB}=3+\eta {c}^{2}-{Z}_{BA},$$

10 $${Z}_{AB}=(9+3\eta +3\eta {c}^{2}+{\eta }^{2}{c}^{2})\frac{{n}_{B}}{Z},$$

where *Z*_*AA*_ and *Z*_*BB*_ are numbers of contacts between particles “A” (case (a) in Fig. 8) and between particles “B” (case (b) in Fig. 8), respectively. *Z*_*AB*_ the number of contacts of particles “A” with a reference particle “B” (cases (c) or reverse (d) in Fig. 8). *c* is the size ratio of the particles, $c={r}_{B}/{r}_{A}$, and *Z* is the mean number of contacts per particle in the bulk. According to Bouvard and Lange^50^, *Z* is exactly equal to 6, which is in agreement with the coordination number model proposed by Ouchiyama and Tanaka^51^. Finally, $\eta$ is a factor given by:

11 $$\eta =3+\frac{Z-3}{{n}_{A}+(1-{n}_{A}){c}^{2}},$$

where *n*_*A*_ and *n*_*B*_ are, respectively, the number fractions of particles “A” and “B” in the mixture given by:

12 $${n}_{A}=1-{n}_{B}=\frac{{N}_{A}}{{N}_{A}+{N}_{B}},$$

with *N*_*A*_ and *N*_*B*_, respectively, the number of particles “A” and “B” in the bulk. Another way of calculating the number of contacts per particles was proposed by Chandratilleke *et al*.^52^ by using the coordination number, who also showed that this number is useful to identify the mechanisms of mixing and demixing in particulate systems. The free binding energy, or work of adhesion, done to separate unit areas of two surfaces or media from contact to infinity in a vacuum can be related to the adhesion force *F*_*AB*_ between two particles by^53^:

13 $${W}_{AB}={F}_{AB}\frac{{r}_{A}+{r}_{B}}{2\pi ({r}_{A}{r}_{B})},$$

14 $${W}_{AA}={F}_{AA}\frac{1}{2\pi ({r}_{A})}.$$

*F*_*AB*_ and *F*_*AA*_ denote, respectively, the adhesion and cohesion forces existing at a single interparticle contact, *r*_*A*_ and *r*_*B*_ are the radii of particles “A” and “B” respectively, and *W*_*AB*_ refers to the energies per unit area of the “A”–“B” interface. In a bulk of a mixed particles, each reference particle “A” will share *Z*_*AB*_ adhesive bonds and *Z*_*AA*_ cohesive bonds. Thus, the average bonding work per particle of the perfectly mixed system (see Fig. 8(f)) can be obtained by the following equation:

15 $${W}_{m}={W}_{AB}{Z}_{AB}{n}_{A}+{W}_{BA}{Z}_{BA}{n}_{B}+{W}_{AA}{Z}_{AA}{n}_{A}+{W}_{BB}{Z}_{BB}{n}_{B}$$

For a completely segregated system, the number of adhesive bonds is very low comparing to the number of cohesive bonds (see Fig. 8(e)), and therefore the former can be neglected. We can then write the average bonding work of the segregated system as follows:

16 $${W}_{s}={Z}_{AA}{W}_{AA}{n}_{A}+{Z}_{BB}{W}_{BB}{n}_{B}.$$

Here, for a segregated system (Fig. 8(e)), *Z*_*AA*_ = *Z*_*BB*_ = *Z* = 6. The difference of work Δ*W* = *W*_*m*_ − *W*_*s*_ gives the average cohesive work change (i.e., energy change per contact area) per particle, when the system goes from a segregated to a mixed state. Notice that for a monodisperse system, if *F*_*AB*_ = *F*_*AA*_, Δ*W* is equal to zero.

## Supplementary information

Appendixes A, B and C

File with a power point containing videos of the segregation of monodisperse and bidisperse systems
