# Supplementary material for: Cohesion-driven mixing and segregation of dry granular media
Source: Sci Rep. 2019 Sep 17;9:13480. doi: 10.1038/s41598-019-49451-z (PMC6748908; doi:10.1038/s41598-019-49451-z)
Supplement: Supplementary file 1 — Appendixes A, B and C [file 41598_2019_49451_MOESM1_ESM.pdf]

Cohesion-driven mixing and segregation of dry granular media

Ahmed Jarray, Hao Shi, Bert J. Scheper, Mehdi Habibi and Stefan Luding

Appendix A

Figures 1, 2 and 3 show the raw data of the mixing index versus time obtained from image post-processing without smoothing. In all cases, the mixing index increases and exhibits high fluctuations in the first few rotation of drum, then after 20 seconds, the fluctuations amplitude decreases, as the system flows steadily and the final mixing state is reached.

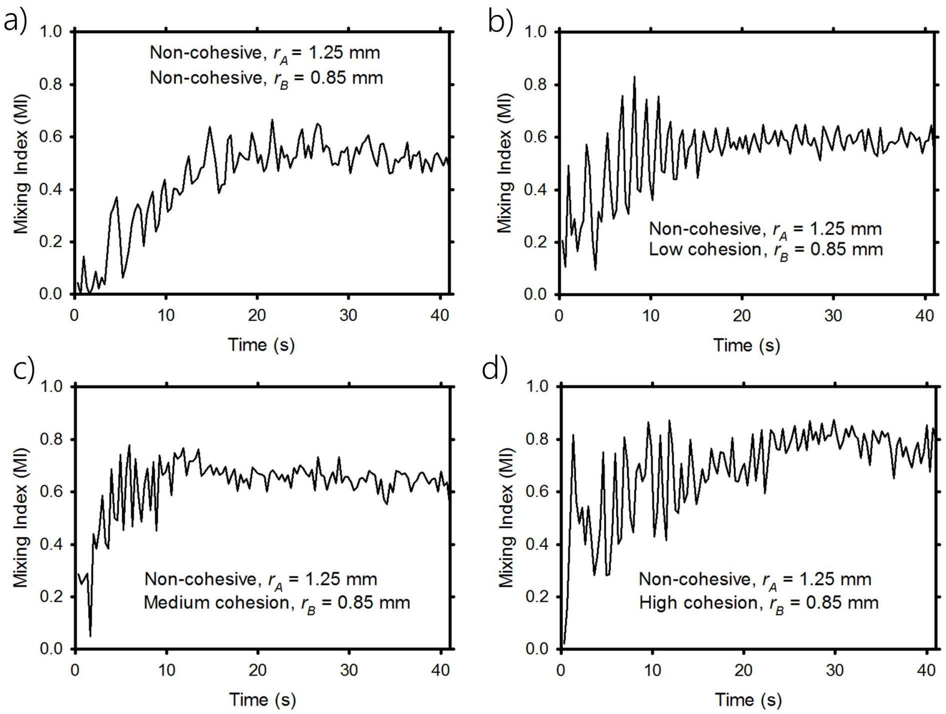

Figure 1. Mixing index as a function of time for for the combination of particles of 0.85 mm and 1.25 mm for different cohesive forces.

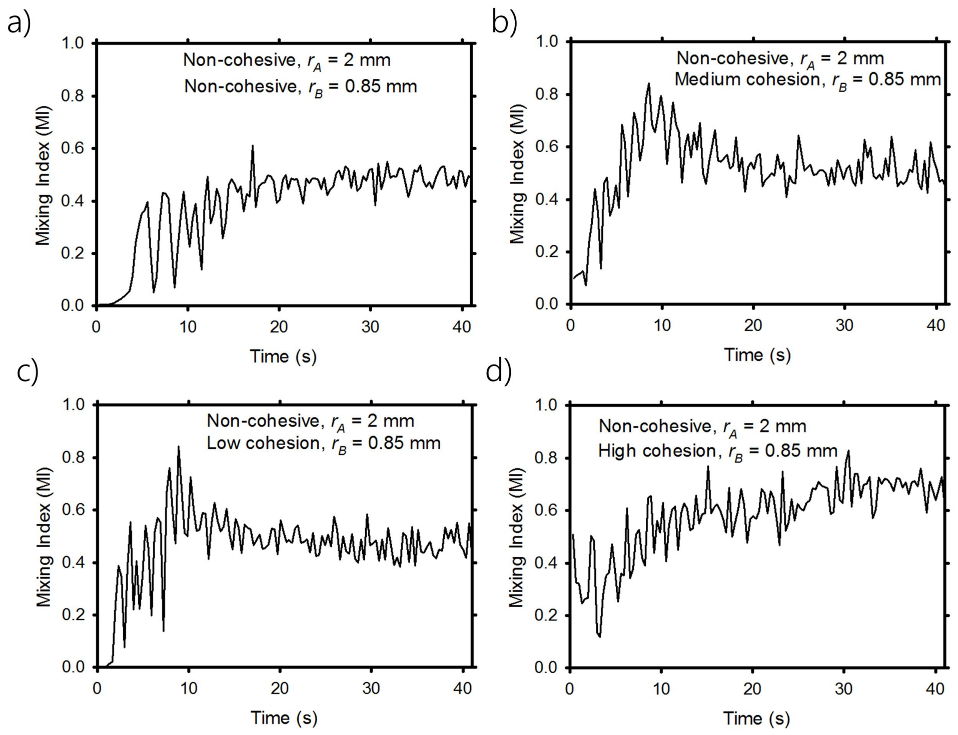

Figure 2. Mixing index as a function of time for for the combination of particles of 0.85 mm and 2 mm for different cohesive forces.

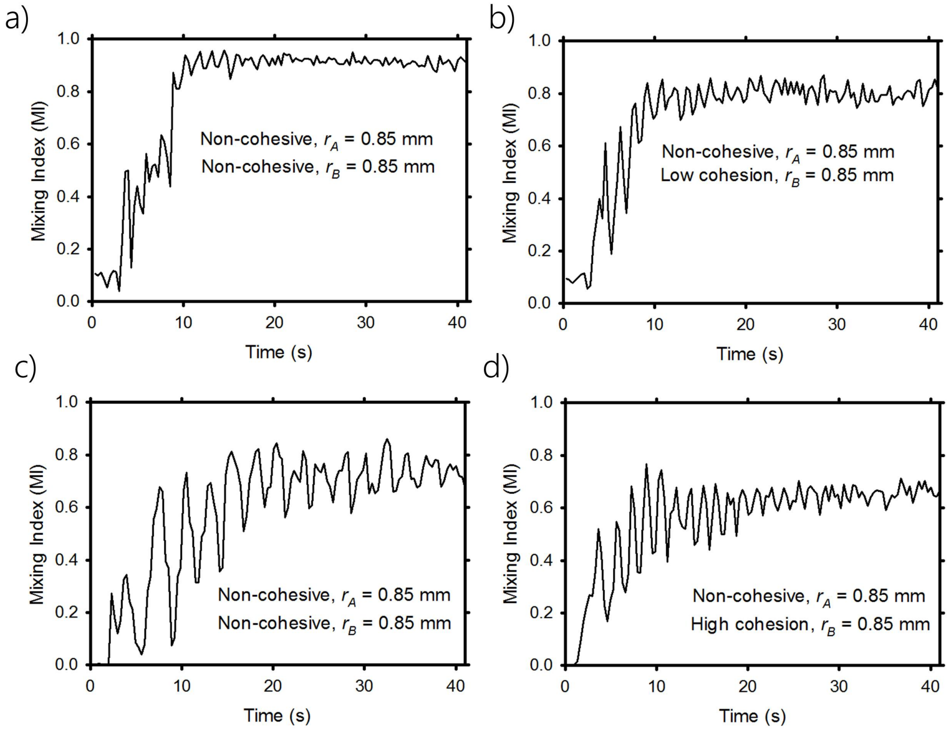

Figure 3. Mixing index as a function of time for monosized particles of 0.85 mm radius and for different cohesive forces.

Appendix B

Fig. 4 shows the granular Bond number, Bo_g, plotted against the critical Bond number, Bo_g^c. The inclined red dashed line where, $Bo_g = Bo_g^c$ delimits the segregation and the mixed zones. It is expected that when $Bo_g > Bo_g^c$, the size of the small clustered particles becomes larger than the individual large particles, causing segregation of the bidisperse system.

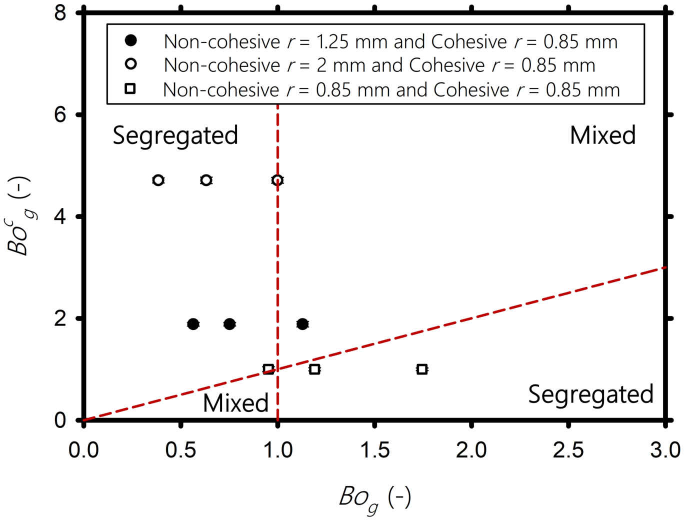

Figure 4. Mixing index as a function of time for monosized particles of 0.85 mm radius and for different cohesive forces.

Appendix C

Figures 5, 6 and 7 show snapshots of the mixing of non-cohesive black particles with transparent particles of radius 0.85 mm, at times 1, 5 and 35 seconds, respectively.

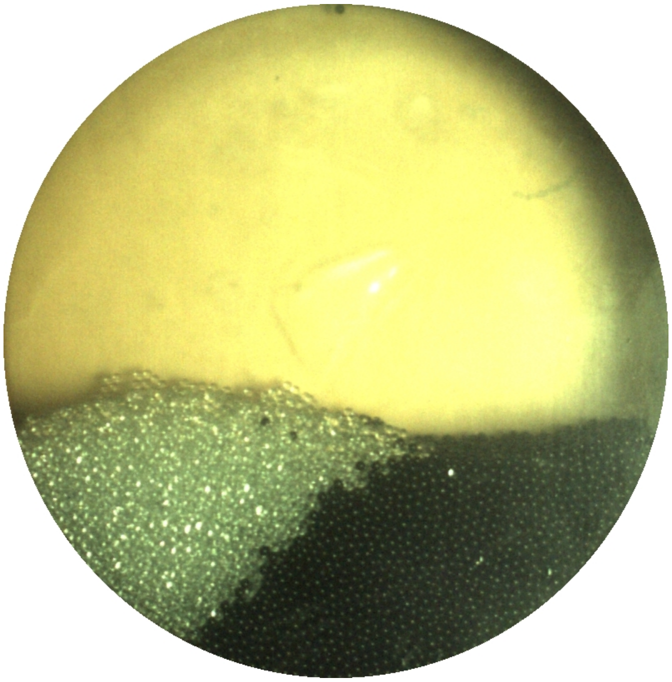

Figure 5. Mixing of monosized particles, black = non-cohesive and transparent = cohesive, time = 1 s.

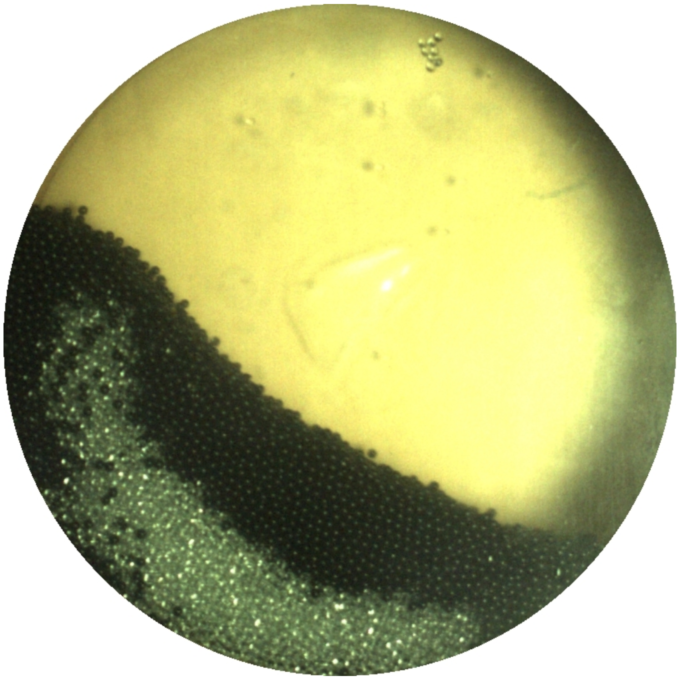

Figure 6. Mixing of monosized particles, black = non-cohesive and transparent = cohesive, time = 5 s.

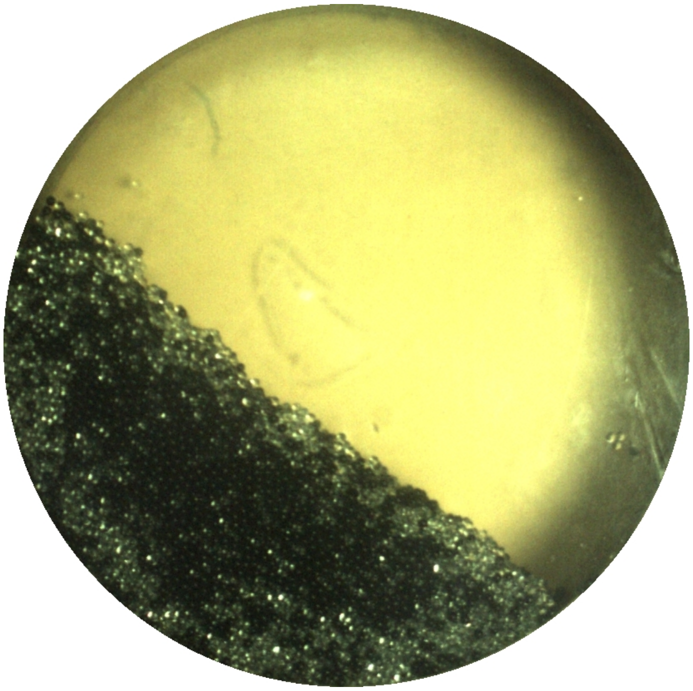

Figure 7. Mixing of monosized particles, black = non-cohesive and transparent = cohesive, time = 35 s.
